# Comprehensive Analysis of RNA-Seq Gene Expression Profiling of Brain Transcriptomes Reveals Novel Genes, Regulators, and Pathways in Autism Spectrum Disorder

**DOI:** 10.3390/brainsci10100747

**Published:** 2020-10-17

**Authors:** Md Rezanur Rahman, Maria Cristina Petralia, Rosella Ciurleo, Alessia Bramanti, Paolo Fagone, Md Shahjaman, Lang Wu, Yanfa Sun, Beste Turanli, Kazim Yalcin Arga, Md Rafiqul Islam, Tania Islam, Ferdinando Nicoletti

**Affiliations:** 1 Department of Biotechnology and Genetic Engineering, Faculty of Biological Sciences, Islamic University, Kushtia 7003, Bangladesh; rezanur12@yahoo.com (M.R.R.); taniaislam1304@gmail.com (T.I.); 2 Department of Biochemistry and Biotechnology, Khwaja Yunus Ali University, Enayetpur, Sirajganj 6751, Bangladesh; 3 IRCCS Bonino-Pulejo, 98123 Messina, Italy; m.cristinapetralia@gmail.com (M.C.P.); rossella.ciurleo@irccsme.it (R.C.); alessia.bramanti@irccsme.it (A.B.); 4 Department of Biomedical and Biotechnological Sciences, University of Catania, 95124 Catania CT, Italy; paolofagone@yahoo.it; 5 Department of Statistics, Begum Rokeya University, Rangpur 5404, Bangladesh; shahjaman_brur@yahoo.com; 6 Cancer Epidemiology Division, Population Sciences in the Pacific Program, University of Hawaii Cancer Center, University of Hawaii at Manoa, Honolulu, HI 96813, USA; lwu@cc.hawaii.edu (L.W.); ysun@cc.hawaii.edu (Y.S.); 7 College of Life Science, Longyan University, Longyan 364000, China; 8 Fujian Provincial Key Laboratory for the Prevention and Control of Animal Infectious Diseases and Biotechnology, Longyan 364000, China; 9 Department of Bioengineering, Marmara University, Istanbul 34722, Turkey; beste.turanli@marmara.edu.tr (B.T.); kazim.arga@marmara.edu.tr (K.Y.A.); 10 School of Biomedical Sciences, Faculty of Health, Institute of Health and Biomedical Innovation, Queensland University of Technology (QUT), Brisbane 4059, QLD, Australia; rafiqulislambd7@gmail.com; 11 Department of Pharmacy, Faculty of Biological Science and Technology, Jashore University of Science and Technology, Jashore 7408, Bangladesh

**Keywords:** autisms spectrum disorders, meta-analysis, RNA-sequencing, transcriptomes, protein–protein interaction, molecular pathways

## Abstract

Background: Autism spectrum disorder (ASD) is a neurodevelopmental disorder with deficits in social communication ability and repetitive behavior. The pathophysiological events involved in the brain of this complex disease are still unclear. Methods: In this study, we aimed to profile the gene expression signatures of brain cortex of ASD patients, by using two publicly available RNA-seq studies, in order to discover new ASD-related genes. Results: We detected 1567 differentially expressed genes (DEGs) by meta-analysis, where 1194 were upregulated and 373 were downregulated genes. Several ASD-related genes previously reported were also identified. Our meta-analysis identified 235 new DEGs that were not detected using the individual RNA-seq studies used. Some of those genes, including seven DEGs (*PAK1, DNAH17, DOCK8, DAPP1, PCDHAC2,* and *ERBIN, SLC7A7*), have been confirmed in previous reports to be associated with ASD. Gene Ontology (GO) and pathways analysis showed several molecular pathways enriched by the DEGs, namely, osteoclast differentiation, TNF signaling pathway, complement and coagulation cascade. Topological analysis of protein–protein interaction of the ASD brain cortex revealed proteomics hub gene signatures: *MYC, TP53, HDAC1, CDK2, BAG3, CDKN1A, GABARAPL1, EZH2, VIM,* and *TRAF1*. We also identified the transcriptional factors (TFs) regulating DEGs, namely, *FOXC1, GATA2, YY1, FOXL1, USF2, NFIC, NFKB1, E2F1, TFAP2A, HINFP*. Conclusion: Novel core genes and molecular signatures involved with ASD were identified by our meta-analysis.

## 1. Introduction

Autism spectrum disorder (ASD) is a severe neurodevelopmental disorder that limits communication, social interactions, and repetitive behaviors are observed in ASD-affected people. There is no cure for this disease yet. The Centers for Disease Control (CDC) has reported that 1 in 54 children has ASD in the US, according to Autism and Developmental Disabilities Monitoring (ADDM) Network (Data published in 2016) [1]. The pattern of prevalence of ASD has been predicted to rise in the coming years. The pathophysiological mechanisms of ASD are very complex and multiple etiological processes are involved. Although genetic heritability is considered to be the principal cause of the disease, various immunological, neurological and environmental factors are involved in ASD. The pathophysiology of non-genetic forms of ASD has yet to be determined. Thus, identification of specific genes and molecular pathways is an unmet challenge, which will uncover potential mechanisms and new avenues to the development of treatment targets for ASD.

Changes in gene expression are widely studied to characterize various human diseases and successfully used to predict molecular and cellular processes in complex diseases [2,3]. Recently, some studies have been focused ASD pathogenesis to decode gene expression signatures specific for ASD. Previous studies found that several candidate genes demonstrated differential expression of ASD patients in brain and blood, as compared to healthy controls [4,5,6,7,8,9]. Despite some important data having been obtained from previous analyses [4,10], we here applied a System Biology approach to further deepen the understanding of the pathophysiological mechanisms in ASD. In this study, two independent publicly available ASD brain studies were used to perform a meta-analysis aimed at identifying novel ASD candidate genes. Additionally, functional enrichment, protein–protein interaction, DEGs-transcription factors, and diseasome analyses were carried out to shed light on potential mechanisms of ASD pathogenesis.

## 2. Materials and Methods

### 2.1. Acquisition of the Transcriptomics Data

**Figure 1:**
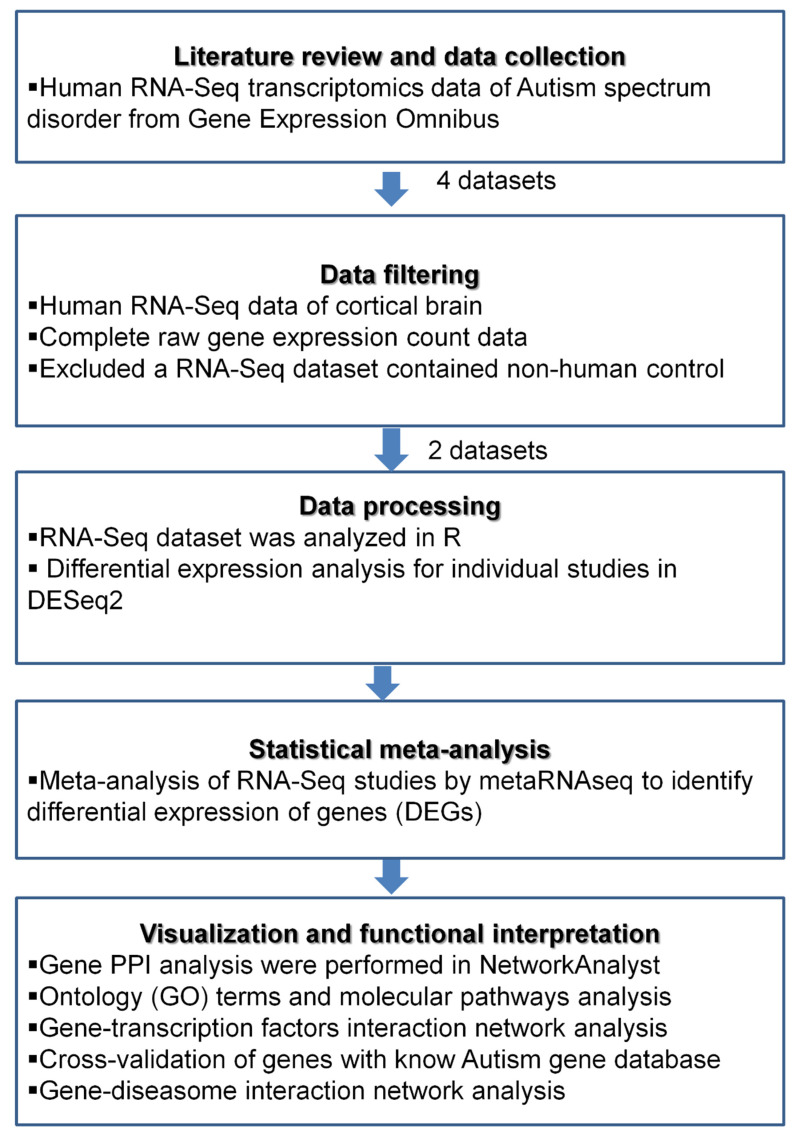
Workflow of data selection and meta-analysis. The Gene Expression Omnibus was searched for human post-mortem RNA-Seq gene expression profiling datasets of brain cortex from autism spectrum disorder patients. Functional analysis was performed on the data obtained from the meta-analysis of the selected datasets.

We searched the big transcriptomics database Gene Expression Omnibus (GEO) in March 2020 to collect the RNA-Seq datasets of human ASD brain samples [11]. We focused on human RNA-Seq data. The criteria used for the selection of the datasets were: (1) human RNA-Seq data of brain cortex; (2) complete raw gene expression count data; (3) the tissues should be from the brain cortex; (4) excluded non-human samples. The detailed procedures are described in Figure 1. Finally, two RNA-Seq transcriptomics datasets from ASD brain cortex region were chosen for this study. The description and characteristics of the data are presented in Table 1. The GSE64018 included transcriptomes from post-mortem brain tissues from 12 ASD and 12 healthy controls, that were age and sex matched. The brain samples from superior temporal gyrus representing Brodmann areas BA41/42/22 were dissected, without harming the gray matter, from all cortical layers. The GSE30573 dataset contains gene expression profiling of post-mortem brain tissues from three ASD samples and three healthy controls from the brain cortex. The detailed demographic data of the samples included in this study is presented in Supplementary Tables S1 and S2.

### 2.2. Data Processing and Differential Expression Analysis of Individual Datasets

The differential expression of individual RNA-Seq dataset was analyzed in R using the package DESeq2 [12]. We used the filtering criteria to include genes that based on the mean of normalized counts as the filter statistic using independent filtering function in DESeq2 with default parameters. We used MetaRNASeq [13] package to perform Fisher’s combined probability test for meta-analysis. Fisher’s combination of *p*-values from independent datasets are combined via the following formula [13,14]:

$$F_{g}=-2\sum_{t=1}^{T}\ln\left(P_{gt}\right)$$

where $P_{gt}$ is the raw *p*-value obtained from gth gene for study t. For independent *p*-values the values of $F_{g}$ lies between 0 to +∞ and it follows Chi-squared distribution with 2*T* degrees of freedom. It produces larger values corresponding to smaller values of $P_{gt}$ and smaller values corresponding to larger values of $P_{gt}$. Therefore, the larger values of $F_{g}$ indicates the rejection of null hypothesis. *p*-values were adjusted by Benjamini–Hochberg false discovery rate (FDR).

### 2.3. Functional Enrichment Analysis

Functional annotation of the identified differentially expressed genes was performed using the clusterprofiler R package [15]. An adjusted *p*-value < 0.05 was considered for the selection of the enriched Gene Ontology (GO) terms and pathways.

### 2.4. Protein–Protein Interaction Analysis

We built a brain protein–protein interactome (PPI) network of the proteins encoded by the DEGs using NetworkAnalyst—an online web tool for data analysis and visualization [16]. The zero-order network was constructed. The hub proteins were selected based on a degree > 15.

### 2.5. Transcriptional Regulators of the DEGs

The network of interactions between DEGs-TFs was constructed using JASPAR database built in NetworkAnalyst [16]. The top 10 identified TFs were considered as principal transcriptional regulators of DEGs.

### 2.6. In Silico Cross-Validation and Gene Disease Association Network Analyses

We cross-checked the identified DEGs found in our meta-analysis with data from SFARI Gene 2.0, a community-driven knowledgebase for the autism spectrum disorders [17]. A gene-disease association network was also built using the DisGeNet database [18], implemented in NetworkAnalyst. DisGeNET is a wide database comprising links from numerous sources that include the complex biological features of disorders. Gene-disorder interaction with specific DEGs was analyzed via a NetworkAnalyst to identify related disease and chronic conditions. Top pathways were detected based on degree measure [19].

## 3. Results

### 3.1. Detection of Differentially Expressed Genes in Brain Cortex via Meta-Analysis of RNA-Seq Transcriptomics

**Figure 2:**
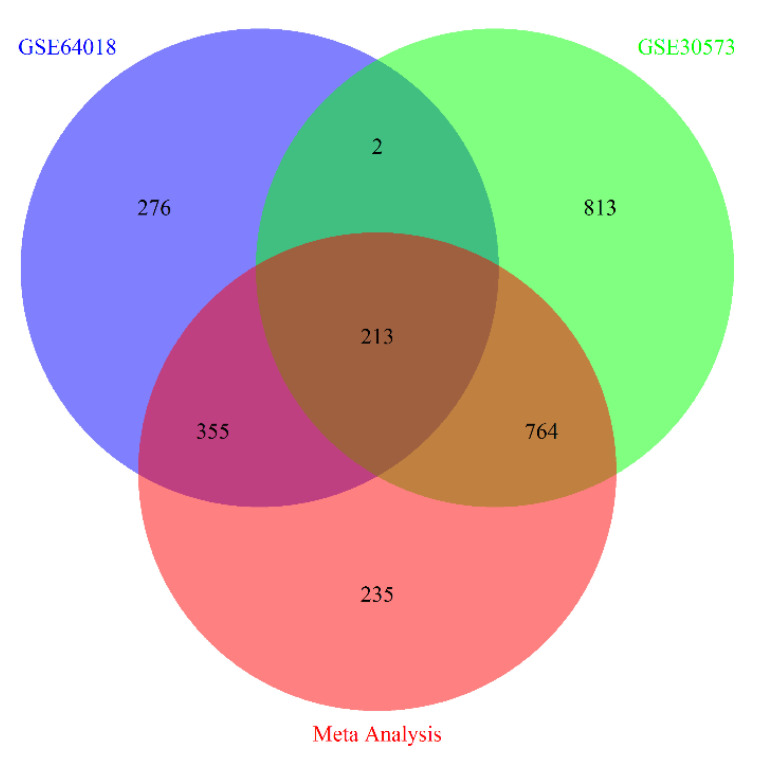
Venn diagram showing the number of differential expressed genes from individual studies and meta-analysis.

We investigated the gene expression profile of the brain cortex of 15 ASD cases and 15 controls (Table 1, Tables S1 and S2). The ASD gene expression profile was obtained by performing a meta-analysis of the selected RNA-Seq studies. Based on the chosen statistical criteria—adjusted *p*-value < 0.05 and log2FC ≥ 1—we detected 1567 differentially expressed genes (DEGs), with 373 downregulated and 1194 upregulated genes in ASD compared to controls (Table S3). The counts of the common genes shared among individual studies/dataset and the meta-analysis are shown as Venn diagram (Figure 2). We identified 65 DEGs that were already known to be causal genes in ASD, from the SFARI autism research database. Among the DEGs identified in the meta-analysis, 235 DEGs were not identified by the analysis of the individual datasets. The top 50 DEGs, sorted by *p*-value, are presented in Table 2. Among these DEGs, seven overlapped with already known causal genes in ASD.

### 3.2. Data Visualization and Functional Interpretation

**Figure 3:**
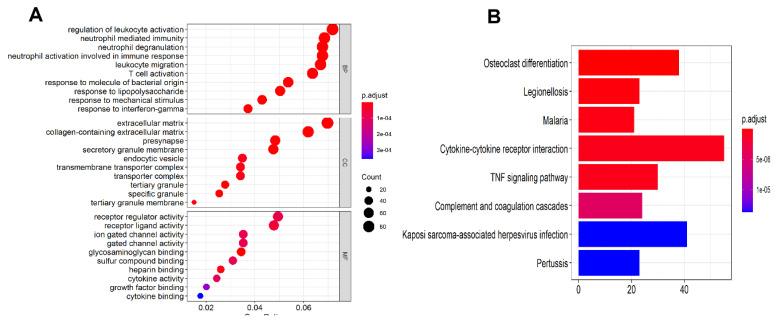
(**A**) Dot plot showing the Gene Ontology (biological process, cellular process, molecular function) analysis for the differentially expressed genes (DEGs) identified in the meta-analysis. Dots are color-coded from blue to red based on the adjusted *p*-value. The size of the dots is proportional to gene count. (**B**) Bar plot shows KEGG molecular pathways enriched by the DEGs (*p*-value <0.05). Bars are color-coded from blue to red based on the adjusted *p*-value.

To decipher the biological involvement and the potential molecular mechanisms underlying the pathogenesis of ASD, we performed GO and Pathway analysis on the DEGs. The GO analyses showed biological process (BP) in the ASD cortex were mainly involved in inflammatory response and immune systems, in particular, regulation of leukocyte activation, leukocyte migration, neutrophil degranulation, and leukocyte migration (Figure 3A). The cellular components encoded by the DEGs were mainly extracellular matrix, secretory granule membrane, presynapse (Figure 3A). The mainly molecular functions (MF) were glycosaminoglycan binding, heparin binding, cytokine activity, ion gated channel activity enriched by the DEGs (Figure 3A).

The pathway analyses revealed that osteoclast differentiation, cytokine–cytokine receptor interaction, TNF signaling pathway, complement and coagulation cascade, pertussis, malaria, legionellosis, Kaposi sarcoma-associated herpesvirus infection were significantly enriched among the DEGs, as shown in Figure 3B.

### 3.3. Hub Proteins: Protein Interactome Analysis

**Figure 4:**
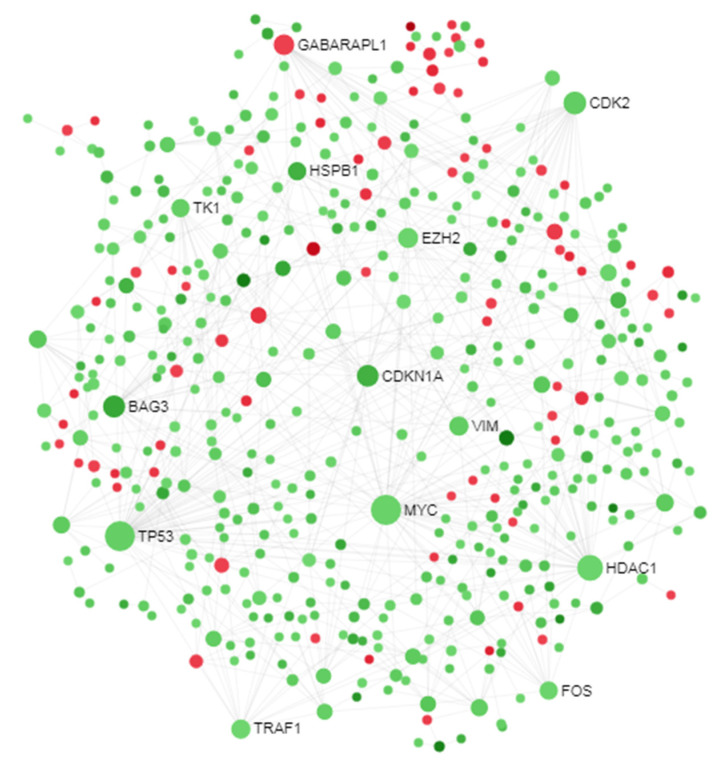
PPI subnetwork analysis of differentially expressed genes (DEGs). Circles represent nodes and grey lines represent interaction partners of the DEGs. The green nodes represent upregulated DEGs, while the red nodes represent downregulated DEGs.

We performed brain protein–protein interaction analysis of the DEGs identified by the meta-analysis using the brain (cortex) protein interactome data. We reconstructed a subnetwork around DEGs encoded protein in dataset or tissue (Figure 4). The first order subnetwork built using DEGs had 6231 nodes and 15,330 edges contained 946 seed nodes. Then, a zero-order network was reconstructed which had 471 nodes, 760 edges and 471 seeds (Figure 4). The degree-based topological analysis of the network revealed 10 hub genes (*MYC, TP53, HDAC1, CDK2, BAG3, CDKN1A, GABARAPL1, EZH2, VIM*, and *TRAF1*). Table 3 shows the details topological parameters, roles, and molecular significance of the hub proteins.

### 3.4. Regulatory Signature: DEGs-TFs Interaction Network

**Figure 5:**
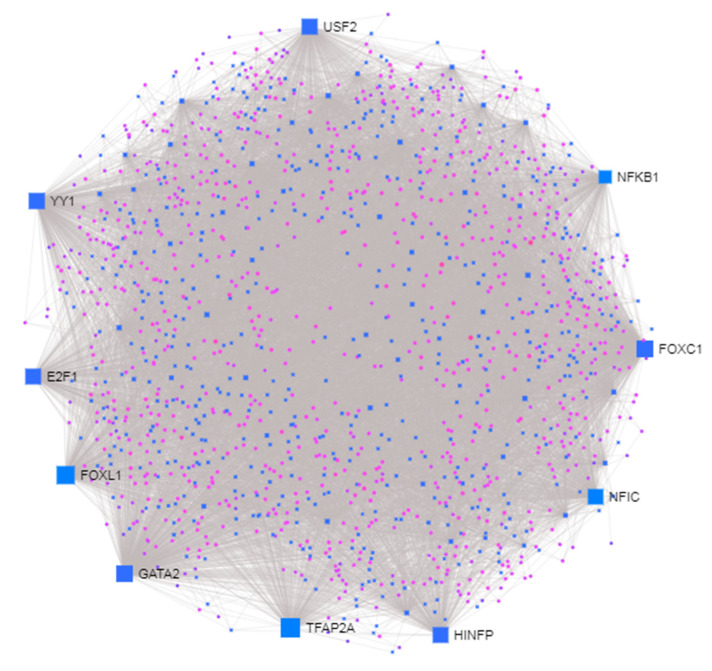
Network constructions of differentially expressed genes and transcription factors. Pink indicates genes and blue squares represent transcription factor. The larger blue squares show the hub transcription factor. The grey lines represent interaction.

A network-based approach was employed to construct a DEGs-TFs interaction network, to identify potential TFs involved in the modulation of the DEGs. *FOXC1, GATA2, YY1, FOXL1, USF2, NFIC, NFKB1, E2F1, TFAP2A, HINFP* were detected as TFs regulating DEGs (Table 4 and Figure 5).

### 3.5. Cross-Validation and DEGs–Disease Association Network

We next compared the identified 1567 DEGs with known ASD genes from SFARI database and found that 65 overlapping genes, namely, *RIT2, PAK1, HCN1, CECR2, KCNC1, DNAH17, KRT26, RBFOX1, SLC9A9, OXTR, DAPP1, CAPN12, KIF14, ATP1A1, DISC1, PBA2, CADPS2, FRMPD4, ATP2B2, MSR1, PCDH15, GABRG3, GRIN2A, PTPRC, DOCK1, C4B, SAMD11, THBS1, LRP2,ERBIN, SLC7A7, STXBP1, SHOX, PLAUR, KANK1, ATP1A3, DPP4, DOCK8, FBXO40, SNAP25, RIMS3, CADPS, GABRB2, ITPR1, SEZ6L2, PCDHAC2, VSIG4, CTNNA3, SLC4A10, VDR, SYT1, CDH22, IL1R2, SCN2A, BCAS1, GFAP, PRKAR1B, SERPINE1, HLA-B, SCN8A, ITGB3, PRICKLE1, RELN, KCNJ15, ARHGEF10*.

**Figure 6:**
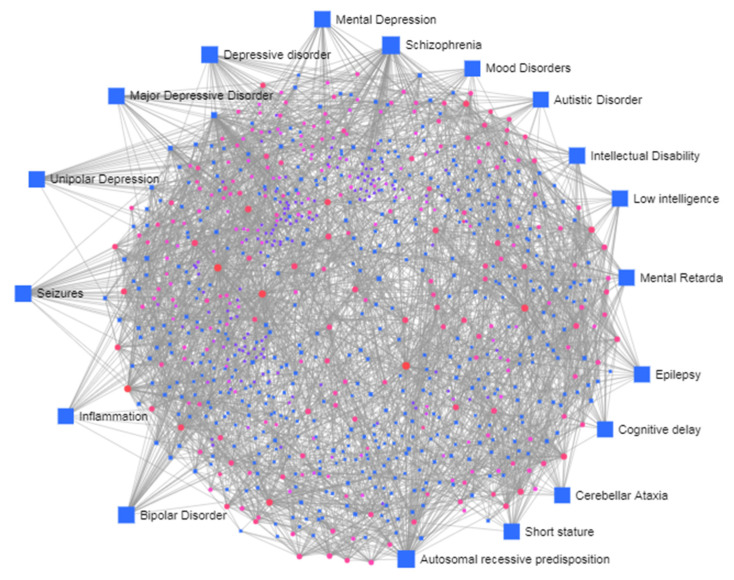
Network constructions of differentially expressed genes and transcription factors. Pink indicates genes and blue squares represent transcription factors. The grey lines represent interaction. The larger blue squares show important disease pathways.

By performing a gene–disease association analysis of the meta DEGs, we found a significant association with diseases such as, Mood Disorders, Autistic Disorder, Intellectual Disability, Unipolar Depression, Mental Retardation, Low intelligence, Global developmental delay, Depressive disorder, Seizures, Bipolar Disorder, Major Depressive Disorder, Mental Depression, Epilepsy, Cognitive delay, Cerebellar Ataxia, Inflammation (Figure 6). Our data suggest that the DEGs identified in ASD may be in common with other neurodevelopmental disorders.

## 4. Discussion

ASD is a prevalent neurodevelopmental disease that is characterized by impaired social communication and repetitive behaviors. To better understand the biological mechanisms of ASD, we decided to perform a comprehensive systems biology analysis of RNA-seq transcriptomics of the ASD brain cortex. With this aim, we performed an integrative RNA-Seq gene expression profiling in cortex to identify transcriptional gene signatures altered in 15 ASD and 15 controls (Table 1, Tables S1 and S2). From the quantitative meta-analysis, we identified 1567 differentially expressed genes (DEGs), 373 downregulated and 1194 upregulated.

Among the top DEGs, PVALB (encoding for parvalbumin) was identified as one of top DEGs (FDR < 0.05 and logFC = −3.3). Parvalbumin (PV) is a calcium-binding protein expressed in a subset of GABAergic interneurons in the brain. Recently, this population of inhibitory (GABAergic) interneurons, expressing the calcium-binding protein PV (here indicated as Pvalb neurons), has attracted much attention with regard to ASD [34]. Indeed, Pvalb neurons control the coordination of neuronal networks and studies on ASD animal models have shown a reduced number of PV-immunoreactive (PV+) cells. Moreover, mice with reduced (PV+/−) or absent (PV−/−) PV expression levels show behavioral deficits similar to those observed in human ASD patients [35]. On top of that, a decreased number of Pvalb neurons has been also found in ASD post-mortem brains [36]. However, the PVALB gene has not been identified as an ASD risk gene by GWAS studies, suggesting that functional alteration of Pvalb neurons are sufficient to promote the disease, despite the presence of PVALB genetic alterations.

With the present meta-analysis, we were able to detect 235 unique DEGs, not identified by the individual studies (Table 2), supporting the increased statistical power of the meta-analysis approach [13,37]. Seven of these DEGs (*PAK1, DNAH17, DOCK8, DAPP1, PCDHAC2,* and *ERBIN, SLC7A7*), have been reported to be deferentially expressed in ASD. Mutation of *PAK1* has been reported to be involved in intellectual neurodevelopmental disorder [38,39]. Genetic variants in *DNAH17* gene were reported to be associated with ASD [40]. Two separate ASD cases reported a novel repeated duplication of the DAPP1 gene [41]. In another study, two SNPs within *PCDHAC2* gene were associated with ASD [41].

Among the enriched pathways in ASD, we found the osteoclast differentiation pathway, which has been previously associated with ASD. Indeed ASD children have been found to have low bone density and increased risk of fractures [42,43,44,45]. Many factors play a key roles in the establishment and maintenance of bone density suggesting that common factors could be commonly involved in regulating bone density and autism [46]. In addition, osteoclast dysfunction was also observed in another neurodevelopmental/intellectual development disorder, Snyder Robinson syndrome [47]. Another pathway, the TNF signaling pathway, was also significantly deregulated in ASD. Several studies have shown increased levels of TNF in ASD subjects in brain, cerebrospinal fluids and plasma indicating the crucial roles of TNF signaling in ASD [48,49,50]. In line with our observation, other recent studies have highlighted the immunological involvement in the pathogenesis of ASD [51]. Cytokines mainly function as immunological response mediators, but they also have major nervous system associations. They engage in normal neuronal growth and function and may have several neurological consequences for improper actions [52]. In addition, altered cytokine levels are associated with severity symptoms of ASD [53]. In a previous study, the complement protein C3 was identified to be differentially expressed in blood of ASD versus controls [54]. Therefore, the complement pathway might be linked with inflammatory processes underlying ASD. These observations corroborated our findings suggesting that the identified pathways are crucial in development of treatment strategies in ASD, and hence can be exploited to develop new drug targets.

In the present study, we also characterized the potential TFs involved in the regulation of DEGs expression. The *FOXC1* plays a role in normal development of cerebellar and posterior fossa. It has been demonstrated that deletion or duplication of *FOXC1* are associated with cerebellar malformation, suggesting the *FOXC1* dysregulation play role in neurodevelopmental disease [28]. *GATA2* is involved in maintaining the development of GABAergic neurons, movement and neuron-specific gene expression via direct action or control of other GABAergic genes [29]. However, its association with development of ASD is not known yet. *YY1* is widely expressed in brain and have role in development and neurodegeneration. Gabriele et al. showed that both deletions and de novo point mutations affecting *YY1* activity trigger Intellectual Disability syndrome of haploinsufficiency with a wide variety of development and psychiatric comorbidities [30]. Twelve known ASD SNPs are associated with validated TF binding sites of *YY1*, *E2F1* and *USF2* enriched in neurodevelopmental and neuro-psychiatric disorder PPI network [31]. Many of these SNPs are correlated with synaptic transmission. GWAS study revealed genetic variants of NFIC is associated with Alzheimer’s disease [32]. A genome-wide association study identified *NFKB1* to play crucial role in etiology of treatment refractory schizophrenia in the Chinese Han population [33]. The roles of the *TFAP2A* and *HINFP* have not yet been described in other neurodevelopmental disorders.

The topological analysis of the protein–protein interactions is a useful approach to decode the critical signaling molecules in complex disease. The PPI analysis identified hub gene signatures, that included *BAG3, CDK2, CDKN1A, EZH2, GABARAPL1, HDAC1, MYC, TP53, TRAF1* and *VIM*. *BAG3* was identified to be significantly differentially expressed in Schizophrenia by gene and pathways-based meta-analysis of microarray data [55]. Genetic variants of *BAG3* have been reported to be involved in Parkinson’s disease [20]. A recent report revealed that genetic variants of *CDKN1A* have been implicated in ASD in dorsolateral prefrontal cortex region of brain [21]. Another hub protein, *EZH2*, is a chromatin remodeler factor and it plays essential functional roles in nervous system biology, including differentiation, neurological and cognitive development. The genetic variation of *EZH2*-a chromatin remodeling factors is observed in intellectual disabilities and ASD [22]. Li et al. showed aberrant expression of *EZH2* in human embryonic brain suggesting a contributory role of this gene in the etiology of ASD in the Chinese population [23]. Therefore, the hub protein EZH2 may be implicated in ASD, in accordance with these data. Several studies have suggested a role for HDAC1 and *HDAC2* in learning and memory behaviors [24]. Assessment of copy number variations suggested *GABARAPL1* involved in ASD associated pathway [25]. However, differential expression has not been previously reported. However, the roles of these genes in ASD are still unclear. The role of *VIM* and *TRAF1* in ASD pathogenesis is also not known yet. Here, we suggest that further experimental studies are warranted to establish their role as biomarkers in ASD.

Despite the tremendous significance of the present study, several limitations can be noted, in that the two RNA-Seq datasets were obtained at different times and small samples size. Additionally, the racial and ethnic background of the subjects and control did not completely match.

Our study differs significantly from other studies performed on circulating blood cells from ASD toddlers [8]. It is likely that genes that are expressed across many tissues represent major contributors to the heritability of a complex disorders, such as ASD. Given the strong genetic basis of ASD, some dysregulated developmental pathways could continually reoccur in leukocytes and thus could be studied postnatally in tissues that are easily obtained. Blood cells from ASD toddlers show perturbations in several biological processes, that include cell proliferation, differentiation, and microtubules organization, and this is in accordance with some dysregulated processes that have been described in iPSC-derived neural progenitors and neurons from individuals with ASD [8].

However, the study of blood cells cannot provide several pieces of information on dysregulated biological processes that, irrespective of the presence of genetic alterations, occur in ASD brain. One particular example is provided by parvalbumin, which is not a bona fide ASD risk gene, but is significantly altered in a population of inhibitory interneurons, recently recognized to be implicated in ASD manifestations, as previously mentioned [34,35,36].

## 5. Conclusions

In conclusion, the present study aimed at characterizing the whole-genome transcriptomic profile of the brain cortex from ASD patients. Our meta-analysis and systems biology analyses revealed core gene signatures as candidate biomarkers and etiopathogenetic pathways in ASD. A proteomic signature of hub genes (*MYC, TP53, HDAC1, CDK2, BAG3, CDKN1A, GABARAPL1, EZH2, VIM,* and *TRAF1*) was identified as a key driver in ASD. In general, core signatures of DEGs associated with ASD genes, including already known markers of ASD and novel hub genes, were identified. Potential implicated TFs were also discovered. The identified core gene signatures may represent candidate biomarkers and drug targets. For many of the newly identified DEGs in this study, direct associations with ASD has not previously reported. Therefore, experimental research is needed to validate these new DEGs to decipher their biological roles in ASD in order to better understand the perturbed molecular mechanisms. The present study reported novel genes and pathways in ASD, and further investigation is required for validation of hub genes in the clinical setting.

## Figures and Tables

**Table 1:** Characteristics of datasets employed in the study.

GEO Accession	Brain	No of Sample
GSE64018	cortex	Control: 12 ASD:12
GSE30573	cortex	Control: 3 ASD: 3

*GEO: Gene Expression Omnibus*.

**Table 2:** Newly differentially expressed genes newly identified in the meta-analysis.

Ensemble ID	Gene Symbol	FDR	Average log_2_FC	Regulation of Direction
ENSG00000077420	*APBB1IP*	5.92E-06	1.02	Upregulated
ENSG00000134516	*DOCK2*	6.38E-05	1.07	Upregulated
ENSG00000142102	*PGGHG*	0.000223	1.06	Upregulated
ENSG00000146192	*FGD2*	0.000394	1.27	Upregulated
ENSG00000142583	*SLC2A5*	0.000449	1.31	Upregulated
ENSG00000188282	*RUFY4*	0.000537	2.67	Upregulated
ENSG00000053918	*KCNQ1*	0.000553	1.11	Upregulated
ENSG00000123338	*NCKAP1L*	0.000703	1.15	Upregulated
ENSG00000107099	*DOCK8*	0.000823	1.04	Upregulated
ENSG00000249825	*AC012636.1*	0.001274	1.21	Upregulated
ENSG00000128602	*SMO*	0.001487	1.19	Upregulated
ENSG00000213694	*S1PR3*	0.001625	1.06	Upregulated
ENSG00000184574	*LPAR5*	0.001879	1.25	Upregulated
ENSG00000198142	*SOWAHC*	0.002218	1.07	Upregulated
ENSG00000197324	*LRP10*	0.002269	1.22	Upregulated
ENSG00000104903	*LYL1*	0.002295	-1.01	Upregulated
ENSG00000152192	*POU4F1*	0.002449	3.19	Upregulated
ENSG00000188511	*C22orf34*	0.002528	1.09	Upregulated
ENSG00000132561	*MATN2*	0.003122	1.00	Upregulated
ENSG00000137767	*SQOR*	0.003406	1.14	Upregulated
ENSG00000178623	*GPR35*	0.003868	1.10	Upregulated
ENSG00000137693	*YAP1*	0.004274	1.18	Upregulated
ENSG00000187554	*TLR5*	0.004764	1.05	Upregulated
ENSG00000155465	*SLC7A7*	0.005133	1.10	Upregulated
ENSG00000092531	*SNAP23*	0.005308	1.03	Upregulated
ENSG00000183508	*TENT5C*	0.005427	1.61	Upregulated
ENSG00000136732	*GYPC*	0.005534	1.03	Upregulated
ENSG00000258701	*LINC00638*	0.005609	1.23	Upregulated
ENSG00000158516	*CPA2*	0.005742	2.25	Upregulated
ENSG00000135245	*HILPDA*	0.006065	1.25	Upregulated
ENSG00000125398	*SOX9*	0.006225	1.24	Upregulated
ENSG00000142512	*SIGLEC10*	0.006448	1.31	Upregulated
ENSG00000105137	*SYDE1*	0.006757	1.05	Upregulated
ENSG00000084093	*REST*	0.006965	1.20	Upregulated
ENSG00000167393	*PPP2R3B*	0.007156	1.02	Upregulated
ENSG00000174348	*PODN*	0.007183	1.08	Upregulated
ENSG00000143384	*MCL1*	0.007261	1.05	Upregulated
ENSG00000231327	*LINC01816*	0.007409	1.07	Upregulated
ENSG00000168209	*DDIT4*	0.007787	1.04	Upregulated
ENSG00000127418	*FGFRL1*	0.007833	1.05	Upregulated
ENSG00000225032	*AL162586.1*	0.008133	1.14	Upregulated
ENSG00000101916	*TLR8*	0.008409	2.09	Upregulated
ENSG00000155926	*SLA*	0.008477	1.07	Upregulated
ENSG00000121933	*TMIGD3*	0.008491	1.16	Upregulated
ENSG00000205336	*ADGRG1*	0.008543	1.04	Upregulated
ENSG00000101057	*MYBL2*	0.0087	3.23	Upregulated
ENSG00000165806	*CASP7*	0.008879	1.09	Upregulated
ENSG00000223764	*LINC02593*	0.008879	1.67	Upregulated
ENSG00000104689	*TNFRSF10A*	0.009434	1.10	Upregulated
ENSG00000225684	*FAM225B*	0.009746	1.62	Upregulated

FDR: False Discovery Rate; FC: Fold Change.

**Table 3:** Hub proteins identified from protein–protein interaction network analysis of corresponding proteins encoded by the differentially expressed genes obtained in the meta-analysis.

Gene Symbol	Description	Regulation	Degree	Biological Significance	Reference
*BAG3*	BAG cochaperone 3	Up	23	Parkinson’s disease	[20]
*CDK2*	Cyclin dependent kinase 2	Up	25	Involved in cell cycle regulation	GeneCard
*CDKN1A*	Cyclin dependent kinase inhibitor 1A	Up	22	Implicated in ASD in dorsolateral prefrontal cortex region of brain	[21]
*EZH2*	Enhancer of zeste 2 polycomb repressive complex 2 subunit	Up	18	Genetic variation of EZH2-a chromatin remodeling factors is observed in intellectual disabilities and ASD; EZH2 in human embryonic brain suggesting a contributory role of this gene in etiology of ASD in Chinese population	[22,23]
*HDAC1*	Histone Deacetylase 1	Up	35	Several studies have suggested a role for HDAC1 and HDAC2 in learning and memory behaviors	[24]
*GABARAPL1*	GABA Type A Receptor Associated Protein Like 1	Down	19	ASD associated pathway	[25]
*MYC*	MYC proto-oncogene	Up	54	Implicated in tumorigenesis and metabolism	[26]
*TRAF1*	TNF receptor associated factor 1	Up	16	Involved in inflammation and aberrant expression leads to inflammatory disease	[27]
*VIM*	Vimentin	Up	16	Disease involved in VIM is cataract	Genecards database

**Table 4:** Post-transcriptional regulatory biomolecules identified from differentially expressed genes–transcriptional interaction networks.

TFs	Description	Degree	Molecular Significance	Novelty	Reference
FOXC1	forkhead box C1	785	deletion or duplication of FOXC1 are related with cerebellar and cerebellar malformation	Novel	[28]
GATA2	GATA binding protein 2	652	GATA2 is involved in maintaining the development of GABAergic neurons, its association with development of ASD is not known yet.	Novel	[29]
YY1	YY1 transcription factor	413	both deletions and de novo point mutations affecting YY1 activity trigger Intellectual Disability syndrome of haploinsufficiency	Novel	[30]
FOXL1	Forkhead Box L1	353	The role of the TF FOXL1 is not known in neurodevelopmental disorder	Novel	[31]
USF2	Upstream Stimulatory Factor 2	325	USF2 is one of the major TFs that bind in brain.12 known ASD SNPs are associated validated TF binding sites of YY1, E2F1 and USF2 enriched in neurodevelopmental and neuropshiatric disorder	Known	[31]
NFIC	Nuclear Factor I C	305	highly enriched in neurodevelopmental disorder, not known in ASD	Novel	[32]
NFKB1	Nuclear Factor Kappa B Subunit 1	297	identified NFKB1 play crucial role in etiology of treatment refractory schizophrenia in Chinese Han population; not known in ASD	Novel	[33]
E2F1	E2F Transcription Factor 1	287	12 known ASD SNPs are associated validated TF binding sites of YY1, E2F1 and USF2 enriched in neurodevelopmental and neuropsychiatric disorder PPI network. Many of these SNPs are correlated with synaptic transmission.	Known	[31]
TFAP2A	Transcription Factor AP-2 Alpha	263	The role of the TF TFAP2A is not known in neurodevelopmental disorder.	Novel	-
HINFP	Histone H4 Transcription Factor	258	The role of the TF TFAP2A is not known in neurodevelopmental disorder.	Novel	-

## Supplementary Materials

The following are available online at https://www.mdpi.com/2076-3425/10/10/747/s1, Table S1: Demographic description of post-mortem brain tissue (GSE64018) dataset. Table S2: Demographic description of post-mortem brain tissue (GSE30573) dataset. Table S3: A complete list of differential expressed genes identified in meta-analysis of ASD vs. controls.
